# Arctigenin from Burdock Root Exhibits Potent Antibacterial and Anti-Virulence Properties against *Pseudomonas aeruginosa*

**DOI:** 10.4014/jmb.2403.03003

**Published:** 2024-06-17

**Authors:** Abdulrahman E. Koshak, Mahmoud A. Elfaky, Hossam M. Abdallah, Dina A. I. Albadawi, Gamal A. Mohamed, Sabrin R. M. Ibrahim, Abdulrahim A. Alzain, El-Sayed Khafagy, Azza A. H. Rajab, Wael A. H. Hegazy

**Affiliations:** 1 Department of Natural Products and Alternative Medicine, Faculty of Pharmacy, King Abdulaziz University, Jeddah 21589, Saudi Arabia; 2 Centre for Artificial Intelligence in Precision Medicines, King Abdulaziz University, Jeddah, 21589, Saudi Arabia; 3 Department of Chemistry, Preparatory Year Program, Batterjee Medical College, Jeddah 21442, Saudi Arabia; 4 Department of Pharmaceutical Chemistry, Faculty of Pharmacy, University of Gezira, Wad Madani 21111, Sudan; 5 Department of Pharmaceutics, College of Pharmacy, Prince Sattam bin Abdulaziz University, Al-Kharj 11942, Saudi Arabia; 6 Department of Pharmaceutics and Industrial Pharmacy, Faculty of Pharmacy, Suez Canal University, Ismailia 41522, Egypt; 7 Department of Microbiology and Immunology, Faculty of Pharmacy, Zagazig University, Egypt; 8 Pharmacy Program, Department of Pharmaceutical Sciences, Oman College of Health Sciences, Muscat 113, Oman

**Keywords:** Antimicrobial resistance, gram-negative pathogens, traditional foods, quorum sensing

## Abstract

*Arctium lappa* (Burdock) root is used in various culinary applications especially in Asian Cuisine. Arctigenin (ARC) is a polyphenolic compound abundant in the roots of the burdock plant from which it derives its name. The emergence of bacterial resistance is a growing global worry, specifically due to the declining availability of new antibiotics. Screening for the antibacterial candidates among the safe natural products is a promising approach. The present study was aimed to assess the antibacterial activity of ARC against *Pseudomonas aeruginosa* exploring its effect on the bacterial cell membrane. Furthermore, the anti-virulence activities and anti-quorum sensing (QS) activities of ARC were in vitro, in vivo and in silico assessed against *P. aeruginosa*. The current results showed the ARC antibacterial activity was owed to its disruption effect of the cell membrane. ARC at sub-MIC significantly decreased the formation of biofilm, motility, production of extracellular enzymes and in vivo protected mice against *P. aeruginosa*. These anti-virulence activities of ARC are owed to its interference with bacterial QS and its expression. Furthermore, ARC showed mild effect on mammalian erythrocytes, low probability to induce resistance and synergistically combined with antibiotics. In summary, the promising anti-virulence properties of ARC indicate its potential as an effective supplement to conventional antibiotics for treating severe *P. aeruginosa* infections.

## Introduction

Burdock root (*Arctium lappa*) is used in culinary practices around the world, and its applications can vary across different cuisines. Burdock root, known as "gobo" in Japanese, is a popular ingredient in various Japanese, Korean and Chinese dishes. It is often used in stir-fries, salads, and simmered dishes. In some European cuisines, especially in Scandinavia, burdock root has been historically used in traditional dishes. It may be pickled, added to stews, or used in soups [1]. Arctigenin (ARC) is a lignan polyphenolic compound with potential health benefits compound found in various plants, mainly in the seeds of *Arctium lappa* (burdock), from which it derives its name. ARC is also found trace amounts in some fruits, vegetables, nuts and seeds contributing to their overall polyphenolic content [2]. ARC has been studied for its pharmacological properties including antioxidant, immunomodulatory and anti-inflammatory due its ability to modulate inflammatory pathways [2]. Moreover, the potential anticancer properties of ARC have been investigated as a result to its interference with cancer cell proliferation and apoptosis induction [3]. Further health benefits of ARC include potential anti-obesity [4], anti-diabetic [5], and neuroprotective [6] activities. Importantly, ARC has been investigated for its antibacterial [7], antifungal [8], anti-parasitic [9], and antiviral [10] activities.

*Pseudomonas aeruginosa* is a Gram-negative rod motile bacterium thriving in various natural environments, *P. aeruginosa* displays adaptability to diverse conditions, making it a ubiquitous presence in soil, water, and vegetation [11]. Renowned for its versatile pathogenicity, *P. aeruginosa* is a significant opportunistic pathogen [12]. Particularly in healthcare settings, *P. aeruginosa* has emerged as a formidable foe, causing a range of infections in immune-compromised individuals. *P. aeruginosa* is a notorious for instigating respiratory, urinary, and, bloodstream infections, especially in immunocompromised individuals and those with implanted medical devices [13]. *P. aeruginosa* employs a repertoire of virulence factors, including exotoxins, extracellular enzymes, phenazine pigments, biofilm formation, type three secretion system, and quorum sensing (QS), to augment its ability to colonize and inflict damage to host tissues [11]. Furthermore, *P. aeruginosa* can dynamically modulate its surface antigens and resist phagocytosis, evading the host's immune responses [14]. Interestingly, intrinsic resistance mechanisms and the development of acquired resistance pose significant challenges in treating *P. aeruginosa* infections with conventional antibiotics [15].

Quorum sensing (QS) serves as an intricate communication system utilized by bacteria to synchronize and regulate various behaviors contingent on the local population density [13]. This enables bacteria to act in unison, responding to alterations in their environment and enhancing their chances of survival [16]. Bacteria release diminutive signaling molecules known as autoinducers (AIs), primarily acyl-homoserine lactones (AHLs) in Gram-negative bacteria, into their surroundings [17]. Bacteria are equipped with QS receptors, predominantly Lux-type in Gram-negative bacteria, which can perceive the presence and concentration of AIs [13]. When the bacteria reach a critical concentration threshold, they initiate changes in the expression of genes responsible for virulence. QS plays a pivotal role in coordinating pathogenic behaviors such as biofilm formation, production of virulence factors, and the expression of other genes crucial for survival and adaptation [16].

Bacterial resistance to diverse antibiotic classes is extensively documented and constitutes a significant public health challenge with widespread implications [18]. The dwindling availability of novel antibiotics results in a scarcity of effective options, constraining the treatment possibilities for bacterial infections. This limitation may lead to heightened morbidity and mortality associated with challenging-to-treat infections [19]. Antagonizing QS emerges as a promising avenue for developing novel antimicrobial strategies [20]. QS inhibitors have the potential to disrupt the communication process between bacteria, thereby interfering with the signaling molecules that facilitate their coordinated activities. This interference can impede the establishment and progression of infections [20]. By disrupting QS, coordinated behaviors such as biofilm formation, virulence factor expression, and other pathogenic activities can be hindered, rendering bacteria less virulent [21, 22]. In contrast to traditional antibiotics targeting essential bacterial functions, QS inhibitors may exert less selective pressure on bacteria. This reduced pressure could potentially diminish the likelihood of developing resistance, particularly when employed in conjunction with other therapeutic approaches [16, 23]. The synergy of QS inhibitors with existing antibiotics or alternative antimicrobial agents holds promise for augmenting overall treatment efficacy. This combined approach may address the challenges posed by antibiotic-resistant strains and persistent infections [24, 25].

While the QS antagonism holds promise, there is a need to identify effective, safe, and selective QS inhibitors for clinical applications. Both natural products and synthetic compounds can serve as QS inhibitors. Natural compounds derived from plants and other sources, along with synthetically designed molecules, exhibit potential for disrupting QS [25-27]. The incorporation of QS antagonists with traditional antibiotics or other treatment modalities can enhance the overall effectiveness of the treatment strategy [28-30]. The objective of this study was to assess the antibacterial effectiveness of ARC against *P. aeruginosa* by examining its in vitro, in vivo, and in silico anti-virulence and anti-QS activities.

## Materials and Methods

### Isolation of Arctigenin

ARC was isolated from Burdock seed extract purchased from Xi`an Le Sen Biotechnology Co., Ltd., China. The detailed procedures are presented in the supplementary file (Table S1, Figs. S1 and S2).

### Determination of Minimum Inhibitory Concentrations (MICs)

To determine the minimum inhibitory concentrations (MICs) of ARC and the tested antibiotics against *P. aeruginosa* (ATCC 27853), the broth microdilution method was employed following the protocols outlined by the Clinical Laboratory and Standards Institute [26]. Briefly, 2-fold serial dilutions of ARC were performed, and 100 μl aliquots were transferred to the microtiter plates to be mixed with equal volume of *P. aeruginosa* suspensions with approximate density 1 × 10^6^ CFU/ml. After 18 h. incubation at 37°C, the wells were examined for growth, and the MIC was recognized as the lowest concentration that disallowed visible growth.

### Effect on Cell Membrane

The impact of ARC on *P. aeruginosa* was evaluated using the membrane permeability assay as described in supplementary data [31]. Propidium iodide (PI) was utilized to evaluate the impact of ARC on cell membrane permeability, employing Triton X-100 as positive control and non-treated bacteria as negative control.

### Effect on Proton Motive force (PMF)

To evaluate the effect of ARC on the proton motive force (PMF) of *P. aeruginosa*, 3,3'-Dipropylthiadicarbocyanine Iodide (Disc-3(5)) (Catalog number: 22076-AAT, Strateck, UK) was utilized as described in supplementary data [32].

### Effect on Bacterial Growth at Sub-MIC

The turbidities of fresh cultures were adjusted to the equivalent of 0.5 McFarland standard and then cultivated in the presence or absence of ARC at 1/2 MIC or 1/4 MIC. Viable bacterial counts were performed [33].

### Assay of Biofilm Formation

Biofilm formation was assessed using crystal violet method in the presence or absence of ARC at 1/2 MIC or 1/ 4 MIC concentrations as described in supplementary data [34, 35].

### Effect on Bacterial Motility

LB agar plates provided with ARC at 1/2 MIC or 1/4 MIC were stabbed with 5 μl of freshly prepared overnight *P. aeruginosa* cultures [36]. Negative controls involved the inoculation of untreated bacteria, and the zones of motility were measured in millimeters.

### Protease Assay

The protease production was quantified in *P. aeruginosa* in the presence or absence of ARC at 1/2 MIC or 1/4 MIC as described in supplementary data [37, 38].

### Hemolysins Assay

The production of hemolysin was evaluated in the presence or absence of ARC at 1/2 MIC or 1/4 MIC concentrations as described in supplementary data [24].

### In vivo Mice Protection

The mice protection assay was conducted to assess the ability of ARC at 1/4 MIC to mitigate the pathogenesis of *P. aeruginosa* as described earlier [39, 40]. The fresh *P. aeruginosa* cultures were grown in the absence or presence of the ARC at 1/4 MIC (O.D600 0.4). A total of twenty-five three-week-old Mus musculus mice were divided into five groups, with each group consisting of five mice. Two groups were designated as negative controls and did not undergo any inoculation or intra-peritoneal (IP) injections. Instead, they received sterile PBS injections. The third group was served as positive control and injected (IP) with DMSO-treated *P. aeruginosa*. The fourth group was injected (IP) with *P. aeruginosa* treated with ARC at 1/4 MIC. The micés survival was monitored continuously for six consecutive days.

### Resistance Induction Test

A resistance induction study was performed to test the probability of ARC to develop resistance [41]. Briefly, tested bacterial strains were cultured and the initial MICs of ARC or ciprofloxacin as control antibiotic were determined. Then, the bacteria were passaged to fresh LB containing ARC or ciprofloxacin at 1/2 MIC, and the MICs were measured again. This procedure was repeated daily and changes in MICs were recorded.

### Effect on Red Blood Cells (RBCs)

The hemolysis assay was performed to evaluate the impact of ARC on the hemolytic activity of mammalian erythrocytes, which served as an indicator of its safety [41]. Suspensions of fresh red blood cells (RBCs) in PBS were prepared at a concentration of 8% (v/v). ARC was then added to the RBC suspensions at various concentrations: 2048 μg/ml, 1024 μg/ml, 512 μg/ml, 256 μg/ml, 128 μg/ml, 64 μg/ml, and 32 μg/ml. The mixtures were incubated at a temperature of 37°C for a duration of 1 h, followed by centrifugation. The absorbance of the resulting supernatants was measured at a wavelength of 540 nm. Negative and positive controls were included, utilizing un-hemolyzed erythrocytes and fully hemolyzed erythrocytes achieved by the addition of 0.1% sodium dodecyl sulfate.

### Outcome of Combination with Antibiotics

The checkerboard technique was employed to assess the effect of combining ARC at 1/4 MIC with various tested antibiotics [42]. The outcome of the combination was evaluated using the fractional inhibitory concentration (FIC), which is calculated as the ratio of the MIC of the antibiotic in combination to the MIC of the antibiotic when used alone using the formula MIC of the antibiotic in combination / MIC of the antibiotic alone + MIC of ARC in combination / MIC of ARC alone. Antagonism was observed when the FIC value exceeded 4, while synergy was observed when the FIC value was equal to or less than 0.5. An indifferent effect was considered when the FIC value fell within the range of 0.5 to 4 [43].

### Effect on QS-Encoding Genes

RNA was extracted from fresh overnight cultures of *P. aeruginosa*, including both ARC-treated and untreated samples at a concentration of 1/4 MIC, and the effect of ARC was assessed. The RNA extraction and the primers used [44, 45] and the rt-PCR were previously mentioned [46], are detailed in supplementary data.

### Docking Study

In order to verify the accuracy of our analysis, we gained the X-ray crystallographic structures of three targets associated with *P. aeruginosa*, which are QscR, LasR and PqsR from the Protein Data Bank with the PDB IDs: 3SZT, 2UV0 and 4JVD, respectively. These were prepared via the PPW in Maestro. Throughout the preparation step, the modeled proteins undergo preprocessing to correct the order of all bonds in the structure. Moreover, the models were minimized employing the OPLS3e force field. To locate the binding sites around the current ligands, we applied the Receptor Grid Generation tool. This enabled us to specify the location where the ligand is situated and concentrate our docking simulations on this precise area. ARC and the existing ligands were prepared with the MacroModel module in Maestro to assure that they are suitable for the following docking simulations. We exposed both ARC and the existing ligands to the XP mode of docking in the Glide tool. The docking was conducted in a flexible way, allowing for conformational sampling during the docking phase.

### Statistical Evaluation

All experiments were performed in triplicate, and the results are expressed as the mean ± standard error (SE). The statistical significance of the data was assessed using a two-way analysis of variance (ANOVA) test, with a significance level of *p* < 0.05 indicating statistical significance.

## Results

### Characterization of ARC

The isolated ARC (Fig. S1) was identified by comparing its ^1^H and ^13^C Nuclear Magnetic Resonance (NMR) data with published data [47] (Table S1, Figs. S2 and S3).

### ARC Inhibited *P. aeruginosa* by Disruption of the Cell Membrane

**Fig. 1:**
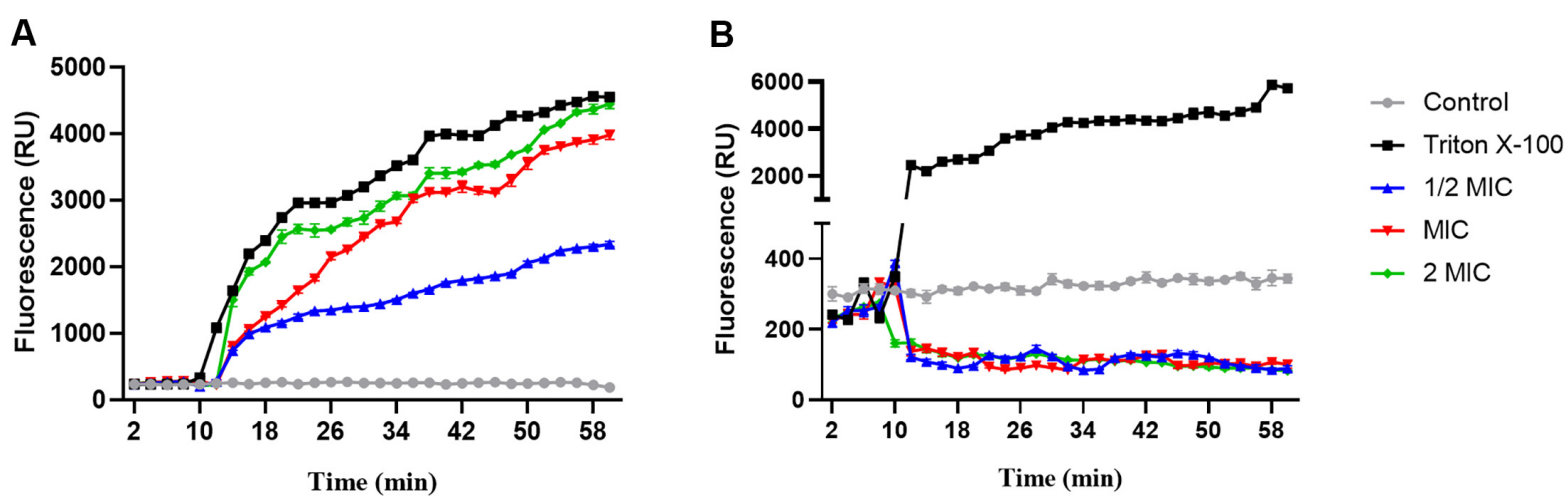
ARC disrupted the *P. aeruginosa* and lowered PMF. (**A**) ARC significantly increased the release of PI indicating the disruption of the cell membrane. (**B**) The PMF was significantly lowered as consequence of the cell membrane damage.

The lowest concentration (MIC) of ARC that inhabited the growth of *P. aeruginosa* was 128 μg/ml. Because of the polyphenolic chemical structure of ARC, it was hypothesized that the ARC antimicrobial activity could be due to the disruption of the cell membrane. In this context the membrane permeability assay was done using PI dye at different concentrations of ARC (Fig. 1A). Upon the cell disruption, the release of PI dye will be increased and hence the fluorescence will be increased. The intensity of PI fluorescence was significantly increased when treating bacterial cells with ARC in a concentration-dependent manner. PMF is typically associated with disruptions or alterations in the integrity of the cell membrane as it is generated by the pumping of protons (H^+^ ions) across the cell membrane, creating a gradient of protons and an electrochemical potential. The Disc3(5) is used to assess the permeability of cell membranes, as it can penetrate cell membranes and emit fluorescence upon binding to intracellular components. Disc3(5) is used in PMF assays as it accumulates in the bacterial membranes in response to the proton motive force. Upon incorporation into the bacterial cell membrane, it exhibits changes in fluorescence intensity based on the membrane potential. Higher membrane potentials lead to increased fluorescence, while decreased potentials result in decreased fluorescence. It is worthy to mention that Triton X-100, the used positive control that disrupts cell membrane the fluorescence of Disc3(5) typically increases. This is because Triton X-100 disrupts the integrity of the cell membrane, allowing more Disc3(5) molecules to penetrate the cells, where they can bind to intracellular components and emit fluorescence. The PMF was assayed at different concentrations of ARC to ensure the cell membrane disruption (Fig. 1B). As predicted, the intensity of Disc3(5) was decreased indicating low PMF as result of cell membrane damage. The experiment was replicated three times, and the statistical significance was determined using two-way repeated measures ANOVA.

### Anti-Virulence Activity of ARC at Sub-MIC

**Fig. 2:**
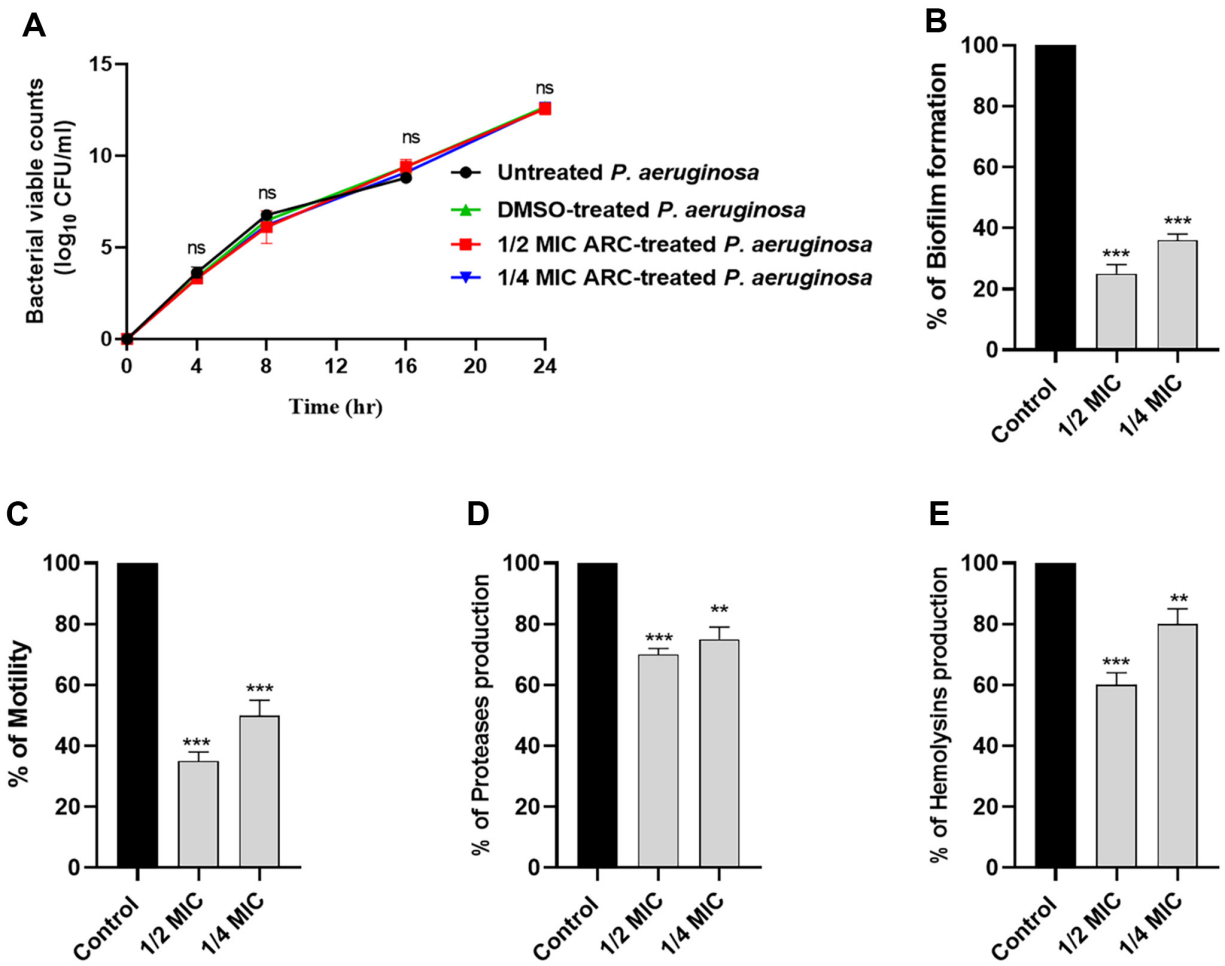
ARC anti-virulence activity. (**A**) viable count of *P. aeruginosa* was done in the presence or absence of ARC at 1/2 MIC and 1/4 MIC. ARC at sub-MIC has no significant effect on bacterial growth. (**B**) The biofilm formation, (**C**) The bacterial motility, (**D**) protease production and (**E**) hemolysins production was significantly decreased in the presence of ARC at sub-MIC. The data are expressed as percent change from un-treated controls. ***: *p* < 0.001, **: *p* < 0.01, and ns: non-significant *p* > 0.05.

To mitigate any potential impact of ARC on bacterial growth, the viability of *P. aeruginosa* cells in the presence of sub-MIC concentrations of ARC (1/2 or 1/4 MIC) was assessed and compared to untreated *P. aeruginosa* (Fig. 2A). The results showed no significant impact of ARC at sub-MIC concentrations on bacterial growth at different time points. The formation of biofilms was assessed using the crystal violet method. ARC demonstrated a significant reduction in biofilm formation, with reductions of 75% and 60% observed at concentrations of 1/2 MIC and 1/4 MIC, respectively (Fig. 2B). Additionally, ARC at 1/2 MIC or 1/4 MIC resulted in approximately 65%and 50% reduction in bacterial motility, respectively (Fig. 2C). *P. aeruginosa* employs various enzymes to facilitate its invasion into host tissues. The production of proteases and hemolysins was evaluated in the presence of ARC at 1/2 MIC or 1/4 MIC (Fig. 2D and 2E). ARC exhibited a significant decrease in the production of both proteases and hemolysins. All results are presented as a percentage change relative to the un-treated bacterial control.

### In vivo Protection of ARC at Sub-MIC against *P. aeruginosa*

**Fig. 3:**
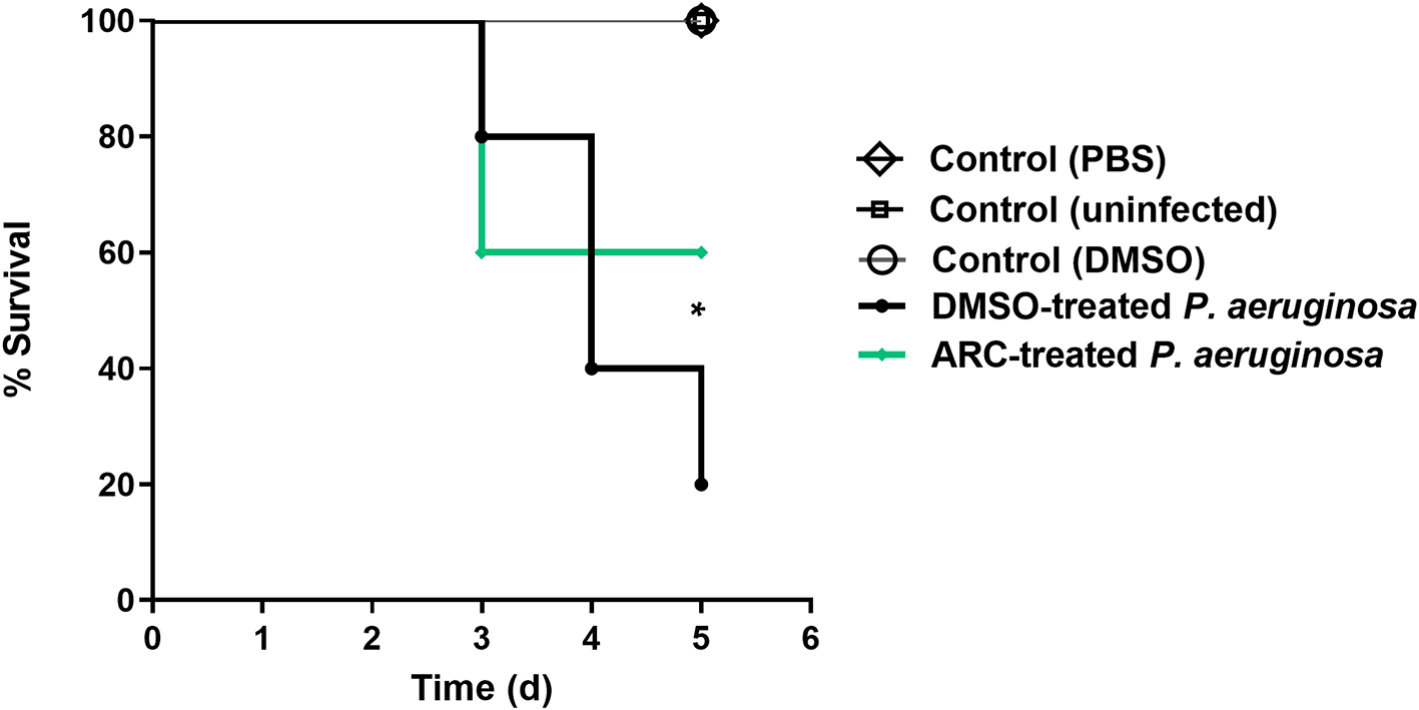
ARC at 1/4 MIC protected mice and significantly diminished the *P. aeruginosa* capacity to induce pathogenesis in mice, Logrank test for trend *p* = 0.015.

The ARC at 1/4 MIC in vivo anti-virulence activity was evaluated by mice protection test against *P. aeruginosa* (Fig. 3). However, there were no deaths recorded in the negative controls, there were 4 deaths recorded in positive control group DMSO-treated *P. aeruginosa*. In the test group injected with *P. aeruginosa* treated with ARC at 1/4 MIC, two deaths were observed. This finding showed the significant ability of ARC at 1/4 MIC to decrease the *P. aeruginosa* capacity to induce pathogenesis in mice (Logrank test for trend, *p* = 0.0115).

### Resistance Development to ARC

**Fig. 4:**
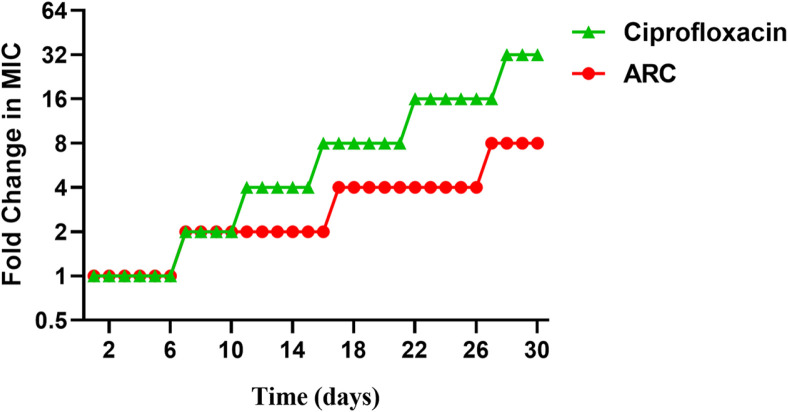
The resistance induction test showed the low probability of *P. aeruginosa* to develop resistance to ARC.

The resistance induction test was conducted to assess the resistance development of *P. aeruginosa* to ARC in comparison to ciprofloxacin (Fig. 4). Interestingly, the antibacterial activity of ARC decreased 8-fold against *P. aeruginosa*, whereas the MIC of the ciprofloxacin activity was 32-fold increased. This result proposes that *P. aeruginosa* could exhibit a low tendency to develop resistance against ARC.

### Safety of ARC to RBCs

**Fig. 5:**
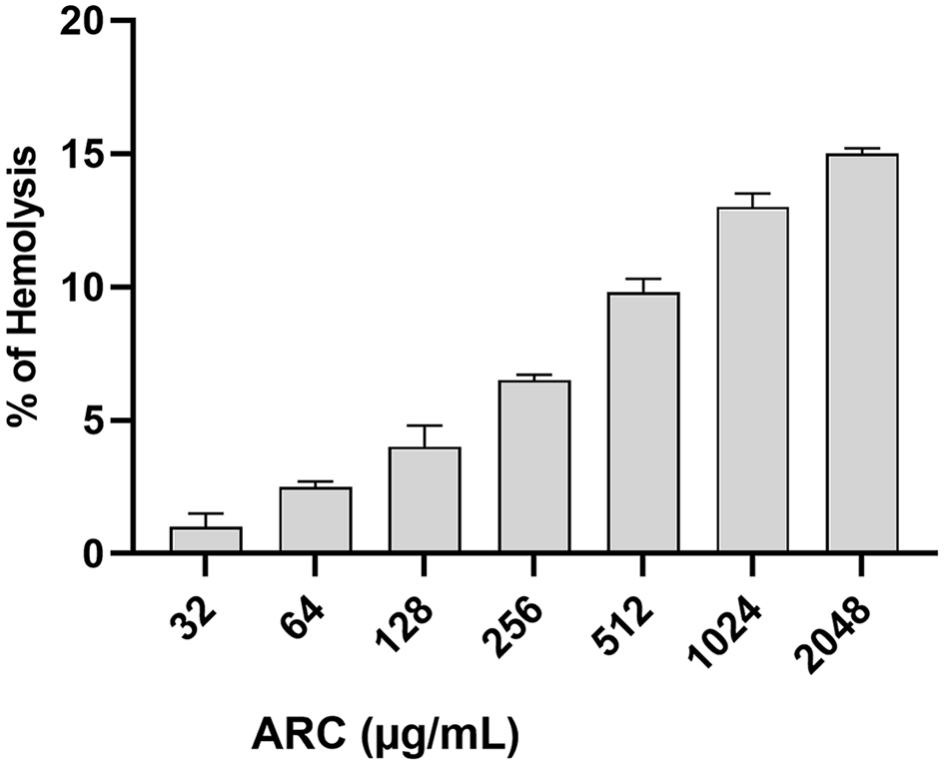
ARC showed a mild effect on RBCs hemolysis indicating its safety.

The hemolytic activity of ARC at different concentrations was assessed (Fig. 5). The hemolytic activity did not exceed 16% at concentration 2,048 μg/ml indicating the ARC safety to the mammalian cells even at concentrations greater than MIC (64 μg/ml).

### Combination Outcome of ARC at Sub-MIC with Antibiotics

The effect of ARC at 1/4 MIC on the potentiation of selected antibiotics in combination with selected antibiotics was evaluated (Table 1). Markedly, the MICs of combinations were reduced showing FIC ≤ 0.5 that indicates the synergistic outcome of combination.

### Anti-QS Activity of ARC

**Fig. 6:**
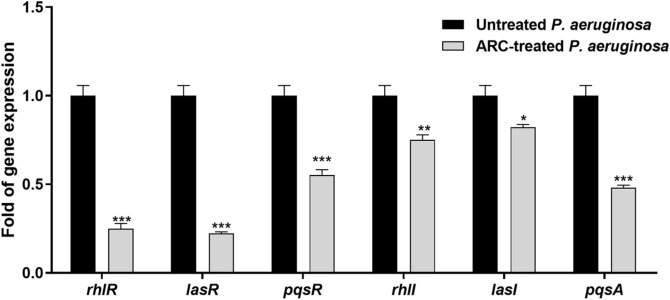
ARC at 1/4 MIC significantly downregulated the expression of QS receptors and autoinducer encoding genes in *P. aeruginosa*. (* *p* < 0.05, ** *p* < 0.01, *** *p* < 0.001).

**ARC lowered the expression of QS-encoding genes.** Reverse transcription polymerase chain reaction (RT-PCR) was employed to assess the impact of ARC at 1/4 MIC on the expression of QS-encoding genes (Fig. 6). A significant reduction in expression of QS-encoding genes was observed upon treatment with ARC at 1/4 MIC. The downregulation effect varies from 3-4-fold as in *lasR*, *rhLR*, and *pqsA* to 1-2-fold in the other genes.

**ARC interaction with QS receptors.** In this study, docking was performed to assess the affinity of ARC towards *P. aeruginosa* by targeting three *P. aeruginosa*-related proteins. The analysis included the investigation of binding modes, interactions, and a comparison of the results with reference ligands. Table 2 displays the diverse binding energies of ARC with QscR, LasR, and PqsR.

**Fig. 7:**
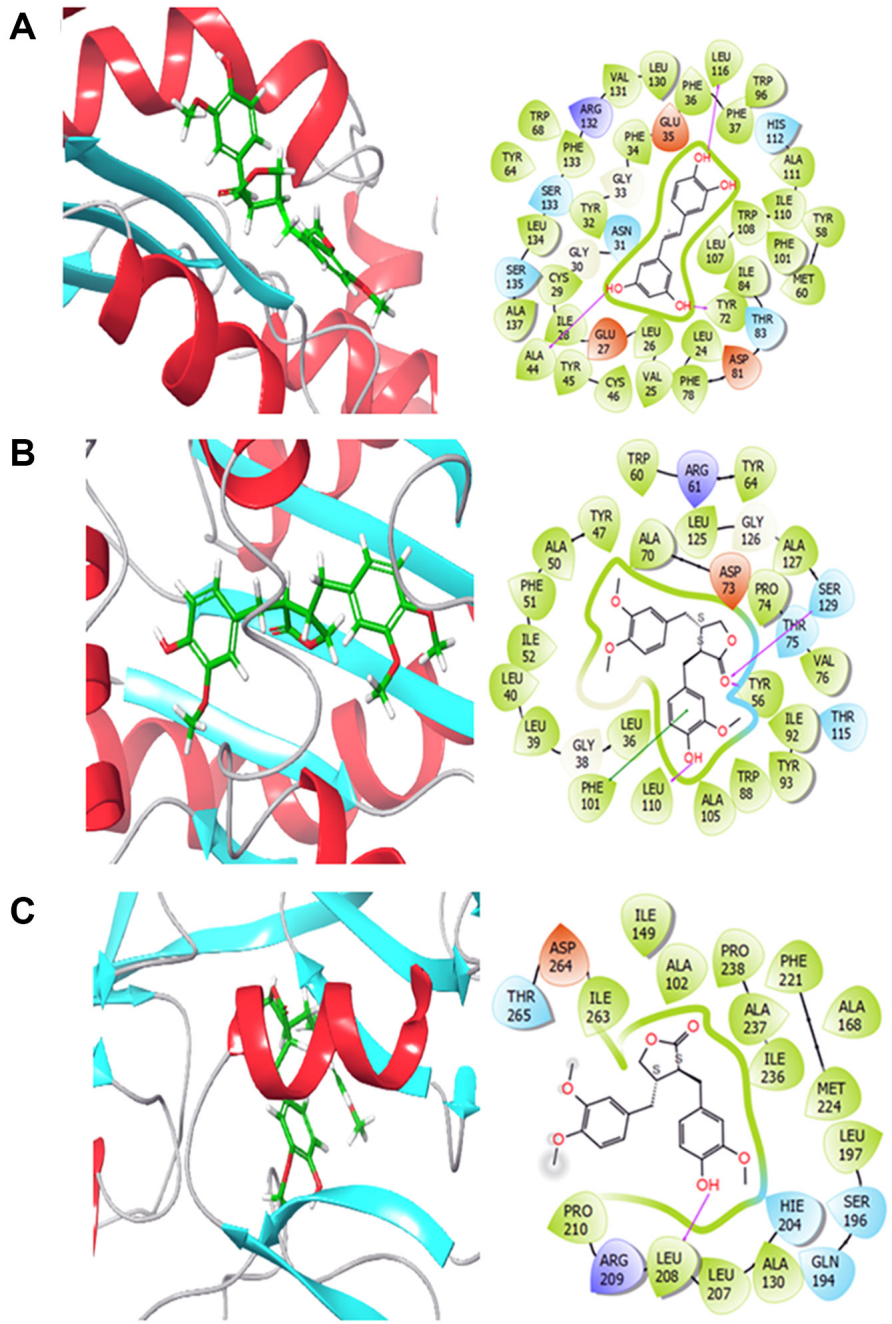
2D and 3D interactions of (+)-Arctigenin bound to various targets related to *P. aeruginosa* (A) QscR, (B) LasR and (C) PqsR.

Regarding the interactions of ARC with these targets (Fig. 7), it was found to form hydrogen bonds with ALA44, TYR72, and LEU116 in QscR, SER129, TYR56, and LEU110 in LasR, and LEU108 in PqsR. Pi-pi interactions were also observed with PHE101 in LasR. Additionally, hydrophobic interactions between ARC and all the targets were detected. These findings suggest stable binding modes for ARC.

Based on the interactions observed and the competitive docking scores obtained in this research for multiple *P. aeruginosa* targets, it can be stated that ARC shows promise as a potential inhibitor of *P. aeruginosa*. The docking data reveal that ARC has the capacity to bind to and interact with essential proteins related to *P. aeruginosa*, including QscR, LasR, and PqsR. These interactions, along with the persistent binding modes found, suggest that ARC could effectively limit the function of these proteins, thereby inhibiting the pathogenicity of *P. aeruginosa*.

## Discussion

ARC is a polyphenolic compound characterized by a dibenzylbutyrolactone skeleton typically derived from the seeds of the traditional herbal medicine, the burdock plant [48]. ARC has undergone investigation for its myriad biological activities and potential therapeutic benefits, encompassing anti-inflammatory, antioxidant, antiviral, and anticancer properties [10, 48-50]. *P. aeruginosa* is known for causing a broad spectrum of infections, producing a huge arsenal of virulence factors and develop resistance to almost all classes of antibiotic [28, 51]. That makes *P. aeruginosa* one of the top listed pathogenic microbes and a good bacterial model to be targeted. The current study aimed to evaluate the antibacterial and anti-virulence activities of ARC against *P. aeruginosa* exploring the mechanisms of action.

Numerous studies have indicated that ARC exhibits antibacterial activity against a wide range of bacteria, encompassing both Gram-positive and Gram-negative strains [7, 52]. The antibacterial actions of ARC are diverse and may encompass mainly the disruption of bacterial cell membranes besides interference with cell wall synthesis and modulation of bacterial cell division [53]. Keeping in mind the polyphenolic nature of ARC; and the fact that the antimicrobial properties of phenolic compounds are frequently ascribed to their influence on bacterial cell membranes [54]. It was hypothesized that ARC antibacterial activity could be due to disruption of bacterial cell membranes. PI is a fluorescent dye that cannot penetrate cell membranes under normal conditions, in live cells with undamaged membranes, PI is restricted from entering. However, when cells undergo membrane damage, as in instances of cell death or injury, the membrane becomes permeable to PI. Consequently, the dye can enter the cells, bind to nucleic acids, and induce a vivid red fluorescence [31]. By quantification of the PI fluorescence intensity, a significant increase was observed in a concentration dependent manner. That indicates the significant damaging effect of ARC on the bacterial cell membrane and this damage increased by increasing the concentration. One of the most important roles of cell membrane is energy production the PMF is formed due to change in the potential across the cell membrane as a result of difference in pH between the intracellular and extracellular [55]. As a consequence to the cell membrane damage, the PMF is dissipated and the capability to produce ATP is lowered [56]. ARC significantly lowered the intensity of a membrane potential sensitive fluorescent dye Disc3(5) as a direct result to the disruption of cell membrane.

Quorum sensing (QS) plays a pivotal role in regulating bacterial virulence by enabling the synchronization of gene expression related to virulence. These genes encode factors that facilitate the establishment and progression of infections [13, 57]. Virulence factors include toxins, adhesins, biofilm formation, and other mechanisms that enhance the ability of bacteria to cause disease [58]. Furthermore, QS enables bacteria to adjust their virulence in response to the host environment [58, 59]. This adaptive response allows bacteria to fine-tune the expression of virulence factors based on the host's defenses and the stage of infection [58]. In this context, targeting specific virulence factors regulated by QS, rather than killing bacteria outright, is a strategy for designing anti-virulence therapies [20, 60]. This approach aims to disarm pathogens and render them less harmful without exerting selective pressure for resistance [16, 61].

*P. aeruginosa* utilizes various types of QS signaling molecules mainly acyl-homoserine lactones (AHLs) [17]. *P. aeruginosa* mainly employs multiple QS systems, including Las, Rhl, and Pqs. The Lux-type systems Las system utilizes AHLs to regulate the expression of numerous virulence factors and the Rhl system influences factors involved in motility and biofilm formation [13, 62]. While, the non-Lux-type Pqs system is associated with the production of secondary metabolites, including quinolone signal molecules [13]. In addition to these three systems, there is QscR which sense the LasI autoinducers [63]. Collectively, these systems collaborate to coordinate the production of virulence factors and facilitate the establishment of infection within the host tissues. Our current findings showed a considered ability of ARC to bind to and interact with QS receptors in *P. aeruginosa*, particularly PqsR. Furthermore, the expression of autoinducer synthetase and QS receptors encoding genes were quantified in the presence of ARC. Remarkably, ARC at sub-MIC concentrations significantly reduced the expression of all QS encoding genes. These anti-QS effects of ARC suggest a substantial potential for anti-virulence activity.

The main merit of targeting QS is mitigating bacterial virulence without affecting the bacterial growth. That does not force the bacteria to develop resistance to the anti-virulence agent [60, 64]. To avoid any effect of ARC on bacterial growth; the effect of ARC at sub-MIC (1/2 MIC and 1/4 MIC) was assessed on the bacterial viable counts at different time points. There was no significant effect of ARC on the bacterial growth in comparison to untreated bacteria.

Biofilm formation is a complex and organized process by which groups of bacteria adhere to each other and to surfaces, forming a slimy and protective matrix [65]. This matrix is often composed of extracellular polymeric substances [66]. Biofilms are highly resistant to antimicrobial agents and host immune responses [67]. It is worthy to declare the tight relation between bacterial motility and its ability to produce strong biofilms; it was shown that the motile bacteria could produce strong biofilms in comparison to non-motile mutants [43]. ARC at sub-MIC significantly decreased the production of biofilm and curtailed the *P. aeruginosa* swarming motility. In addition to biofilm formation and bacterial motility, QS controls the expression of diverse extracellular virulence enzymes as proteases and hemolysins [13, 62]. Both proteases and hemolysins play important roles in the establishment and spread of *P. aeruginosa* infection into the host tissue [62]. ARC at sub-MIC significantly decreased the production of hemolysins and proteases. Moreover, ARC at 1/4 MIC significantly decreased the *P. aeruginosa* capacity to induce pathogenesis in mice. All these results could conclude the potential anti-virulence activity of ARC at sub-MIC.

Our investigations were extended to examine the probability of ARC inducing resistance in *P. aeruginosa*. While the MIC of ARC was increased 8-folds in the resistance induction test, the MIC to ciprofloxacin was increased 32-fold, indicating the low propensity of ARC to induce resistance. The effect of ARC at different concentrations on the RBCs hemolysis was employed as indicator for ARC safety. ARC showed mild effect on RBCs do not exceed 3%at MIC and not more than 16% at concentration 2,048 μg/ml (32 MIC). This could indicate the safety of ARC; however, it requires further toxicological and pharmacological studies to determine the proper dose before clinical application. Finally, the ARC at 1/4 MIC was combined with different antibiotics to assess the rational of its use as adjuvant with antibiotics in treatments. The ARC showed synergistic outcome with the tested antibiotics.

## Conclusion

In nutshell, ARC is a polyphenolic compound extracted from burdock and widely used in traditional herbal medicine. ARC acquires diverse anti-inflammatory, anticancer, and other benefits to the health. In the current study, the antibacterial activity of ARC against *P. aeruginosa* has been explored and it is owed to ARC's disruption effect to the bacterial cell membrane. Furthermore, the anti-virulence activity of ARC was unveiled. ARC significantly decreased the biofilm formation, motility and production of proteases and hemolysins. ARC significantly protected mice from *P. aeruginosa*. These anti-virulence activities were attributed to ARC's anti-QS activities by interfering with QS receptors or downregulating the expression of QS-encoding genes. Additionally, ARC showed mild effect on RBCs indicating its safety and showed low probability to induce resistance. ARC synergistically combined with different antibiotics. These results conclude the potential anti-virulence activities of ARC to serve as adjuvant in combination with antibiotics for treatment of aggressive *P. aeruginosa* infections.

## Supplemental Materials

Supplementary data for this paper are available on-line only at http://jmb.or.kr.



## Figures and Tables

**Table 1:** Interaction between ARC and antibiotics.

Antibiotic	*P. aeruginosa*		
	MIC	MIC_ARC_	FIC
Ciprofloxacin	2	0.5	0.265
Cefepime	8	4	0.515
Cefoperazone	32	32	1.01
Imipenem	16	8	0.516
Piperacillin/tazobactam	16	8	0.515

FIC: Fractional inhibitory concentration = MICARC drug in combination/MIC drug alone. The result of the combination may be antagonistic (FIC > 4), indifferent (FIC > 0.5 to 4), or synergistic (FIC ≤ 0.5). MICs and MICARC are in μg/ml.

**Table 2:** XP docking scores of Arctigenin and the co-crystalized references with various targets related to *P. aeruginosa*.

Name	PubChem ID	Docking Scores (kcal/mol)		
		QscR (PDB ID: 3SZT)	LasR (PDB ID: 2UV0)	PqsR (PDB ID: 4JVD)
(+)-Arctigenin	28125531	-2.632	-9.535	-7.175
Co-crystalized reference	-	-9.878	-10.097	-6.698
