# Supplementary material for: Arctigenin from Burdock Root Exhibits Potent Antibacterial and Anti-Virulence Properties against Pseudomonas aeruginosa
Source: J Microbiol Biotechnol. 2024 Jun 17;34(8):1642–52. doi: 10.4014/jmb.2403.03003 (PMC11380511; doi:10.4014/jmb.2403.03003)
Supplement: Supplementary file 1 [file jmb-34-8-1642-supple.pdf]

J. Microbiol. Biotechnol. 2024. 34(8): 1642–1652
https://doi.org/10.4014/jmb.2403.03003

Supplementary Table and Figures

Arctigenin from Burdock root exhibits potent antibacterial and anti-virulence properties against *Pseudomonas aeruginosa*

Abdulrahman E. Koshak [a], Mahmoud A. Elfaky [a,b], Hossam M. Abdallah [a,*], Dina A. I. Albadawi [a], Gamal A. Mohamed [a], Sabrin R. M. Ibrahim [c,d], Abdulrahim A. Alzain [e], El-Sayed Khafagy [f,g], Azza A. H. Rajab [h], Wael A. H. Hegazy [h,i,]

[a] Department of Natural Products and Alternative Medicine, Faculty of Pharmacy, King Abdulaziz University, Jeddah 21589, Saudi Arabia

[b] Centre for Artificial Intelligence in Precision Medicines, King Abdulaziz University, Jeddah, 21589, Saudi Arabia

[c] Department of Chemistry, Preparatory Year Program, Batterjee Medical College, Jeddah 21442, Saudi Arabia;

[d] Department of Pharmacognosy, Faculty of Pharmacy, Assiut University, Assiut 71526, Egypt

[e] Department of Pharmaceutical Chemistry, Faculty of Pharmacy, University of Gezira, Wad Madani 21111, Sudan

[f] Department of Pharmaceutics, College of Pharmacy, Prince Sattam bin Abdulaziz University, Al-Kharj 11942, Saudi Arabia

[g] Department of Pharmaceutics and Industrial Pharmacy, Faculty of Pharmacy, Suez Canal University, Ismailia 41522, Egypt

[h] Department of Microbiology and Immunology, Faculty of Pharmacy, Zagazig University, Egypt

[i] Pharmacy Program, Department of Pharmaceutical Sciences, Oman College of Health Sciences, Muscat 113, Oman.

[*] **Correspondence: Hossam M. Abdallah**. Department of Natural Products and Alternative Medicine, Faculty of Pharmacy, King Abdulaziz University, Jeddah 21589, Saudi Arabia

Email: hmafifi@kau.edu.sa

1.1. Isolation of Arctigenin

Arctigenin was isolated from Burdock seed extract purchased from Xi`an Le Sen Biotechnology Co., Ltd., China. The seed extract (50.0 g) of *Arctium lappa* L. was submitted to vacuum liquid chromatography (VLC) using *n*-hexane, EtOAc, and MeOH to afford n-hexane (3.9 g), EtOAc (16.2 g), and MeOH (23.7 g) soluble fractions. Further, the EtOAc fraction was chromatographed over SiO_2 VLC using *n*-hexane:EtOAc gradients (from 5 to 100%) by increasing 5% EtOAc for each fraction to get 20 subfractions (ALE-1–ALE-20). The subfractions were monitored by TLC plates with an authentic sample of arctigenin (Chengdu Biopurify Phytochemicals Ltd., Chengdu City, China). The subfractions ALE-5 to ALE-9 were gathered and subjected to RP-18 CC (150 g × 100 cm × 3 cm) eluting with MeOH:H_2O gradient to get semi-pure compound. The impure arctigenin was purified over Sephadex LH-20 CC (100 g × 100 cm × 5 cm) using MeOH/$CHCl_3$ (90:10).

1.2. Effect on Cell Membrane

The effect of ARC on *P. aeruginosa* was evaluated using the membrane permeability assay [1]. Overnight cultures were adjusted to an optical density of O.D600 0.4 and then mixed with 5 µg/mL propidium iodide (PI) (CAS 25535-16-4; Sigma-Aldrich, USA) for 20 minutes at 37°C. Subsequently, 50 µL of the cultured mixture was transferred to wells of a black polystyrene microtiter plate, and the fluorescence intensity was measured at an excitation wavelength of 535 nm and an emission wavelength of 620 nm every 2 minutes for a duration of 10 minutes. After this initial measurement, ARC was added at final concentrations of 1/2 MIC, MIC, and 2 MIC, and the fluorescence intensity was recorded every 2 minutes for a total of 60 minutes. A 1% Triton X-100 solution was utilized as the positive control, while non-treated bacterial cells served as the negative control.

1.3. Effect on Proton Motive Force (PMF)

To evaluate the effect of ARC on the proton motive force (PMF) of *P. aeruginosa*, 3,3'-Dipropylthiadicarbocyanine Iodide (Disc3(5)) (Catalog number: 22076-AAT, Strateck, UK) was utilized [2]. In summary, overnight cultures were centrifuged and then resuspended in HEPES buffer to achieve an optical density of O.D600 0.4. One µM of Disc3(5) was added to the bacterial suspensions and incubated for 20 minutes in a dark environment. The fluorescence intensity was measured at excitation and emission wavelengths of 660 nm and 675 nm, respectively, every 2

minutes for a total of 10 minutes using a black polystyrene microtiter plate. Subsequently, ARC was added at final concentrations of 1/2 MIC, MIC, and 2 MIC, and the fluorescence intensity was measured every 2 minutes for a duration of 60 minutes. A 1% Triton X-100 solution was used as the positive control, while non-treated bacterial cells served as the negative control.

1.4. Assay of Biofilm Formation

Biofilm formation was assessed in the presence or absence of ARC at 1/2 MIC or 1/4 MIC concentrations [3]. In brief, overnight cultures were prepared and diluted with 5 mL of normal saline. The solution's turbidity was adjusted to O.D600 0.4. Then, 200 µL aliquots of the bacterial suspensions were transferred into sterile 96-well microtiter plates and incubated at 37°C for 24 h. After incubation, the planktonic bacteria were removed, and each well was rinsed twice with 1 mL of sterile distilled water using pipetting. The plates were allowed to air dry for 15 minutes in a 37°C incubator. Subsequently, 200 µL of a 1% crystal violet (CV) solution (prepared in 100% ethanol) was added to each well and incubated at room temperature for an additional 15 minutes. Following this incubation, the CV solution was discarded, and the plates were rinsed three times with 1 mL of sterile distilled water. The plates were then left to dry at room temperature for 20 minutes. To solubilize the crystal violet, 200 µL of 30% glacial acetic acid was added, and the optical densities were measured at OD590 using a microplate reader. The experiment included positive and negative controls, where a sub-MIC concentration of ciprofloxacin and 1% v/v dimethyl sulfoxide served as the positive and negative controls, respectively.

Protease Assay

The protease production was quantified in *P. aeruginosa* in the presence or absence of ARC at 1/2 MIC or 1/4 MIC [4, 5]. Overnight cultures adjusted to an OD600 of 0.4 were grown in LB broth with or without ARC at 1/2 MIC or 1/4 MIC. The supernatants were collected and combined with a mixture of 2% casein in PBS and NaOH (0.1 M) at pH 7.0, in a 1:1 ratio, and then maintained at 37°C for 15 minutes. The reaction was stopped by adding 2 mL of trichloroacetic acid (0.4 M) and allowing it to react for 25 minutes. After centrifugation, the optical densities of the supernatants were measured at 660 nm.

1.5. Hemolysins Assay

The production of hemolysin was evaluated in the presence or absence of ARC at 1/2 MIC or 1/4 MIC concentrations [6]. Briefly, overnight cultures adjusted to an OD_{600} of 0.4 were grown in LB broth with or without ARC at 1/2 MIC or 1/4 MIC. The supernatant was mixed with a 2% erythrocyte suspension in sterile saline at a 1:1 ratio and incubated for 2 h at 37°C. After centrifugation, the optical densities were measured at 540 nm. Negative control samples with unhemolyzed erythrocytes and positive control samples with fully hemolyzed erythrocytes (achieved by the addition of 0.1% sodium dodecyl sulfate) were included in the analysis.

1.6. In Vivo Mice Protection

The mice protection assay was conducted to assess the ability of ARC at 1/4 MIC to mitigate the pathogenesis of *P. aeruginosa* [5]. The fresh bacterial cultures were allowed to grow in the absence or presence of the ARC at 1/4 MIC (O.D600 0.4). The current study was performed in accordance with the Helsinki Declaration, and all animal procedures used were approved by Zagazig University's Institutional Animal Care and Use Committee (ZU-IACUC), Egypt, with approval number (ZU-IACUC/3/F/264/2023). Three-week-old Mus musculus were distributed into five groups, each containing five mice. The first and second groups served as negative controls and were neither inoculated nor intra-peritoneally (ip) injected; they received sterile PBS injections. The third group were ip injected with DMSO-treated *P. aeruginosa* serving as positive control. The fourth group was ip injected with *P. aeruginosa* treated with ARC at 1/4 MIC. The survival of the mice was monitored over six consecutive days.[5]. The fresh bacterial cultures were allowed to grow in the absence or presence of the ARC at 1/4 MIC (O.D600 0.4). Three-week-old Mus musculus were distributed into five groups, each containing five mice. The first and second groups served as negative controls and were neither inoculated nor intra-peritoneally (ip) injected; they received sterile PBS injections. The third group were ip injected with DMSO-treated *P. aeruginosa* serving as positive control. The fourth group was ip injected with *P. aeruginosa* treated with ARC at 1/4 MIC. The survival of the mice was monitored over six consecutive days.

1.7. Effect on QS-Encoding Genes

RNA was isolated from fresh overnight cultures of *P. aeruginosa*, both treated and untreated with ARC at 1/4 MIC, using an RNA extraction and purification kit provided by Life Technologies (USA). The concentration of the extracted RNA samples was determined using a NanoDrop spectrophotometer (ND-1000, USA), and the samples were stored at -80°C [7]. To normalize the expression levels, the housekeeping gene *ropD* was used as a reference, and the specific primers employed were previously described in published studies [5]. For cDNA synthesis, the cDNA reverse transcriptase kit from Applied Biosystems (USA) was utilized. Subsequently, the synthesized cDNA was amplified using the Syber Green I PCR Master Kit from Fermentas (USA). The amplification process was carried out using the Step One instrument from Applied Biosystems.

Table S1. NMR spectral data of ARC (MeOH, 500 and 125 MHz).

	δ_H (Mult., J (Hz)	δ_C
1	-	-
2	-	180.1
3	2.72 (m)	46.5
4	2.58 (m)	41.1
5	3.94, 4.17 (m)	71.5
1`	-	130.1
2`	6.69 (m)	111.7
3`	-	149.1
4`	-	147.7
5`	6.59 (m)	112.3
6`	6.71 (m)	121.6
7`	2.57, 2.68 (m)	34.5
1``	-	129.3
2``	6.69 (m)	112.2
3``	-	147.6
4``	-	145.0
5``	6.59 (m)	114.7
6``	6.71 (m)	120.7
7``	2.82, 2.9 (m)	34.0
3 OCH_3	3.78, 3.80, 3.81	54.9, 54.9, 55.0

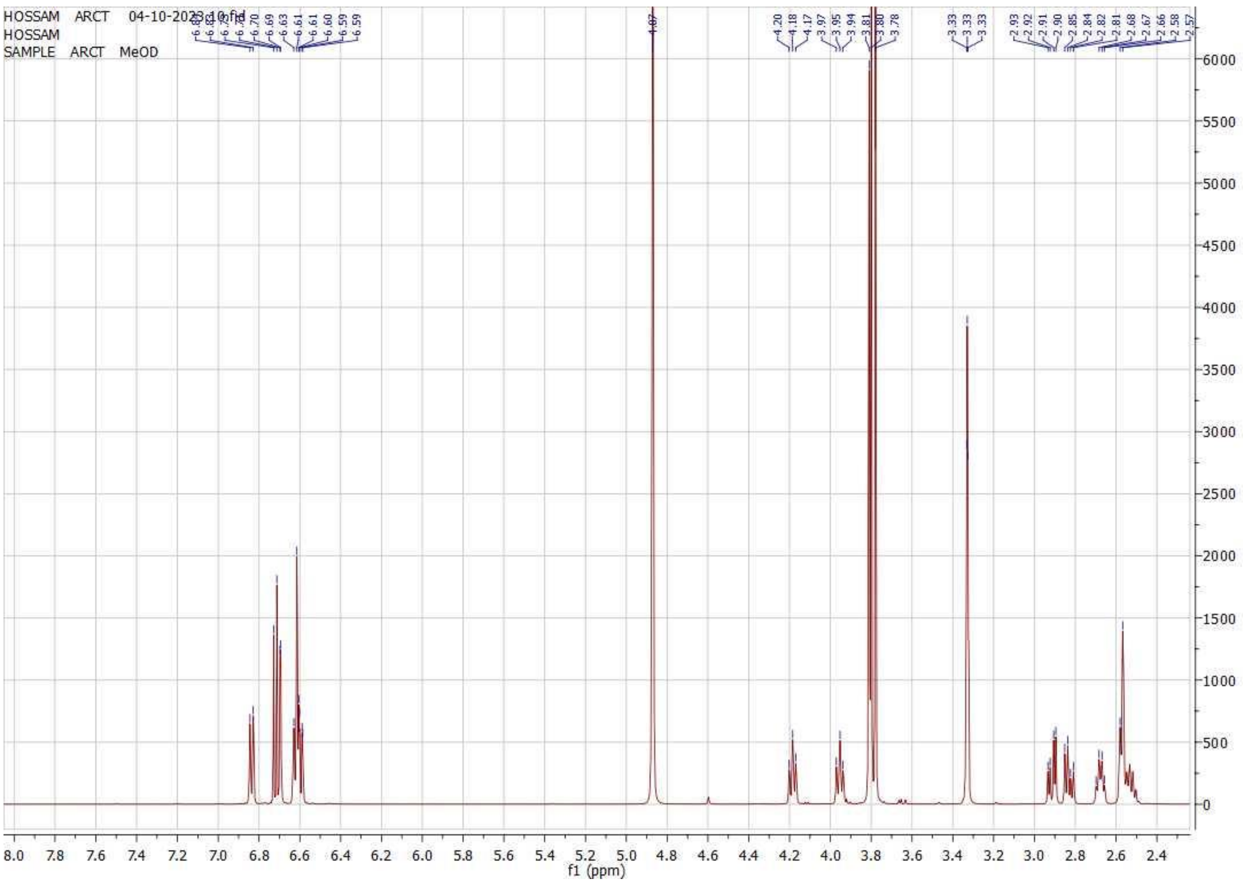

Fig. S1. ^{1}H NMR spectra of Arctigenin (MeOH, 500 MHz).

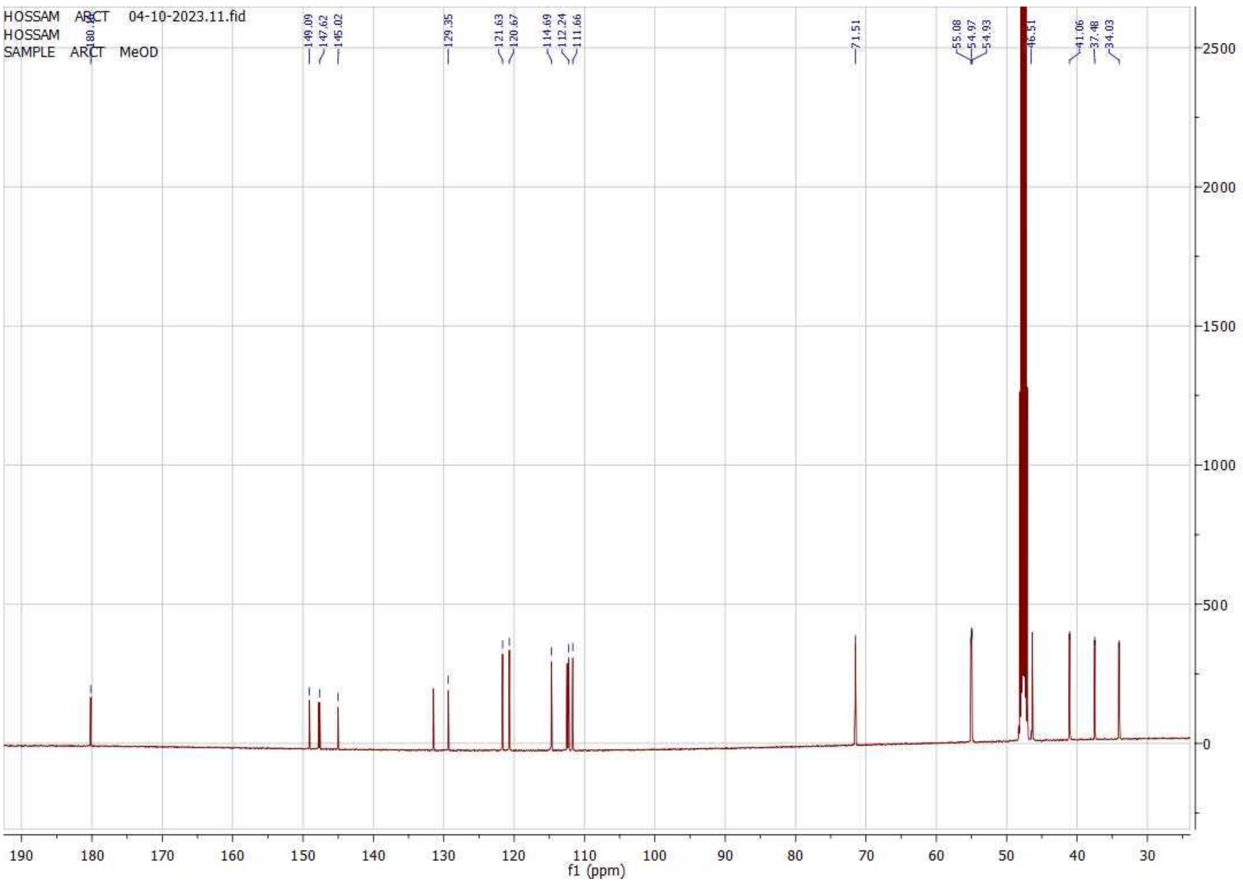

Fig. S2. ^{13}C NMR spectra of Arctigenin (MeOH, 125 MHz).

References

[1] A. Müller, M. Wenzel, H. Strahl, F. Grein, T.N. Saaki, B. Kohl, T. Siersma, J.E. Bandow, H.-G. Sahl, T. Schneider. 2016. Daptomycin inhibits cell envelope synthesis by interfering with fluid membrane microdomains. *Proc. Natl. Acad. Sci. USA* **113**: E7077-E7086.

[2] S.-C. Wu, F. Han, M.-R. Song, S. Chen, Q. Li, Q. Zhang, K. Zhu, J.-Z. Shen, Natural flavones from Morus alba against methicillin-resistant *Staphylococcus aureus* via targeting the proton motive force and membrane permeability. 2019. *J. Agric. Food. Chem*. **67**: 10222-10234.

[3] M.T. Khayat, T.S. Ibrahim, K.M. Darwish, A.N. Khayyat, M. Alharbi, E.S. Khafagy, M.A.M. Ali, W.A.H. Hegazy, H.A. Abbas. 2022. Hiring of the Anti-Quorum Sensing Activities of Hypoglycemic Agent Linagliptin to Alleviate the *Pseudomonas aeruginosa* Pathogenesis. *Microorganisms* **10.**

[4] M.T. Khayat, T.S. Ibrahim, A.N. Khayyat, M. Alharbi, M.A. Shaldam, K.A. Mohammad, E.-S. Khafagy, D.A. El-damasy, W.A.H. Hegazy, H.A. Abbas, 2022. Sodium Citrate Alleviates Virulence in *Pseudomonas aeruginosa*, *Microorganisms* **10**: 1046.

[5] A.A. Saqr, M.F. Aldawsari, E.-S. Khafagy, M.A. Shaldam, W.A.H. Hegazy, H.A. Abbas. 2021. A Novel Use of Allopurinol as A Quorum-Sensing Inhibitor in *Pseudomonas aeruginosa*. *Antibiotics* **10**: 1385.

[6] S. Cavalu, S.S. Elbaramawi, A.G. Eissa, M.F. Radwan, T. S. Ibrahim, E.-S. Khafagy, B.S. Lopes, M.A.M. Ali, W.A.H. Hegazy, M.A. Elfaky. 2022. Characterization of the Anti-Biofilm and Anti-Quorum Sensing Activities of the β-Adrenoreceptor Antagonist Atenolol against Gram-Negative Bacterial Pathogens. *Int. J. Mol. Sci*. **23**: 13088.

[7] M. Askoura, H.A. Abbas, H. Al Sadoun, W.H. Abdulaal, A.S. Abu Lila, K. Almansour, F. Alshammari, E.-S. Khafagy, T.S. Ibrahim, W.A.H. Hegazy. 2022. Elevated Levels of IL-33, IL-17 and IL-25 Indicate the Progression from Chronicity to Hepatocellular Carcinoma in Hepatitis C Virus Patients, Pathogens. **11**: 57.
